# Sorafenib in advanced melanoma: a Phase II randomised discontinuation trial analysis

**DOI:** 10.1038/sj.bjc.6603291

**Published:** 2006-08-01

**Authors:** T Eisen, T Ahmad, K T Flaherty, M Gore, S Kaye, R Marais, I Gibbens, S Hackett, M James, L M Schuchter, K L Nathanson, C Xia, R Simantov, B Schwartz, M Poulin-Costello, P J O'Dwyer, M J Ratain

**Affiliations:** 1 Royal Marsden Hospital, Downs Road, Surrey SMT 5PT, UK; 2 Abramson Cancer Center of the University of Pennsylvania, Philadelphia, PA 19104, USA; 3 Bayer Pharmaceuticals Corporation, West Haven, CT 06516, USA; 4 Department of Medicine, University of Chicago, Chicago, IL 60637, USA

**Keywords:** Sorafenib, multikinase inhibitor, advanced melanoma, V600E *BRAF*, randomised discontinuation trial

## Abstract

The effects of sorafenib – an oral multikinase inhibitor targeting the tumour and tumour vasculature – were evaluated in patients with advanced melanoma enrolled in a large multidisease Phase II randomised discontinuation trial (RDT). Enrolled patients received a 12-week run-in of sorafenib 400 mg twice daily (b.i.d.). Patients with changes in bi-dimensional tumour measurements <25% from baseline were then randomised to sorafenib or placebo for a further 12 weeks (ie to week 24). Patients with ⩾25% tumour shrinkage after the run-in continued on open-label sorafenib, whereas those with ⩾25% tumour growth discontinued treatment. This analysis focussed on secondary RDT end points: changes in bi-dimensional tumour measurements from baseline after 12 weeks and overall tumour responses (WHO criteria) at week 24, progression-free survival (PFS), safety and biomarkers (*BRAF*, *KRAS* and *NRAS* mutational status). Of 37 melanoma patients treated during the run-in phase, 34 were evaluable for response: one had ⩾25% tumour shrinkage and remained on open-label sorafenib; six (16%) had <25% tumour growth and were randomised (placebo, *n*=3; sorafenib, *n*=3); and 27 had ⩾25% tumour growth and discontinued. All three randomised sorafenib patients progressed by week 24; one remained on sorafenib for symptomatic relief. All three placebo patients progressed by week-24 and were re-started on sorafenib; one experienced disease re-stabilisation. Overall, the confirmed best responses for each of the 37 melanoma patients who received sorafenib were 19% stable disease (SD) (ie *n*=1 open-label; *n*=6 randomised), 62% (*n*=23) progressive disease (PD) and 19% (*n*=7) unevaluable. The overall median PFS was 11 weeks. The six randomised patients with SD had overall PFS values ranging from 16 to 34 weeks. The most common drug-related adverse events were dermatological (eg rash/desquamation, 51%; hand-foot skin reaction, 35%). There was no relationship between V600E *BRAF* status and disease stability. DNA was extracted from the biopsies of 17/22 patients. Six had V600E-positive tumours (*n*=4 had PD; *n*=1 had SD; *n*=1 unevaluable for response), and 11 had tumours containing wild-type BRAF (*n*=9 PD; *n*=1 SD; *n*=1 unevaluable for response). In conclusion, sorafenib is well tolerated but has little or no antitumour activity in advanced melanoma patients as a single agent at the dose evaluated (400 mg b.i.d.). Ongoing trials in advanced melanoma are evaluating sorafenib combination therapies.

The incidence of malignant melanoma is rising, and the current treatment options for patients with metastatic disease are limited and noncurative in the majority of cases (Alexandrescu *et al*, 2005; Danson and Lorigan, 2005). According to the American Joint Committee on Cancer, patients with advanced metastatic melanoma (stage IV) have a 5-year survival rate of only 2% (Balch *et al*, 2001).

Increased signalling through the RAF/MEK/ERK pathway, as a result of autocrine stimulation by basic fibroblast growth factor and hepatocyte growth factor, is implicated in melanocytic tumorigenesis (tumour growth, invasion and metastasis) (Satyamoorthy *et al*, 2003). Furthermore, the activity of ERK, which is downstream of RAF, has been shown to increase from early- to advanced-stage melanoma (Satyamoorthy *et al*, 2003). This increased ERK activity may be the consequence of activating *BRAF* mutations, which are present in up to 80% of human melanomas (Davies *et al*, 2002; Chang *et al*, 2004; Garnett and Marais, 2004). The most prevalent oncogenic *BRAF* mutation is the V600E *BRAF* mutation (previous terminology, V599E), which is present in 63% of melanomas (Brose *et al*, 2002). The increased apoptosis, observed in human melanoma cell lines when *BRAF* expression is downregulated using RNA interference, supports a role for oncogenic *BRAF*-driven MEK/ERK overactivation in maintaining the transformed phenotype of malignant melanoma cells (Hingorani *et al*, 2003; Karasarides *et al*, 2004). This observation also suggests that *BRAF* is a rational target for the design of targeted agents to treat melanoma.

The orally administered targeted-agent sorafenib (Nexavar®, Bayer Pharmaceuticals Corporation, West Haven, CT, USA) was originally developed as an inhibitor of the RAF serine/threonine kinases (RAF-1, wild-type BRAF, V600E *BRAF*) (Wilhelm *et al*, 2004). However, results of *in vitro* studies have since shown that sorafenib is a potent multikinase inhibitor, which also targets receptor tyrosine kinases associated with tumour angiogenesis (VEGFR-2, VEGFR-3, PDGFR-*β*) and tumour progression (c-KIT, FLT-3) (Wilhelm *et al*, 2004). Sorafenib has also been shown to inhibit the growth of several human tumour xenograft models by targeting tumour cell proliferation and/or endothelial cell-mediated tumour angiogenesis (Wilhelm *et al*, 2004).

Sorafenib monotherapy has been shown to have a manageable side effect profile in Phase I/II/III studies (Strumberg *et al*, 2003, 2005; Ratain *et al*, 2004, 2006; Awada *et al*, 2005; Escudier *et al*, 2005). The most common toxicities associated with sorafenib are hand-foot skin reaction (HFS), rash and diarrhoea (Strumberg *et al*, 2003, 2005; Ratain *et al*, 2004, 2006; Awada *et al*, 2005; Escudier *et al*, 2005). However, these adverse events are predominantly mild to moderate in severity and easily manageable. The efficacy of sorafenib monotherapy in patients with advanced, refractory renal cell carcinoma (RCC) was first demonstrated in a Phase II randomised discontinuation trial (RDT), which showed a significantly longer progression-free survival (PFS) relative to placebo. Sorafenib received marketing approval in the US in December 2005 for the treatment of advanced RCC, based on the results of the RDT as well as the Phase III placebo-controlled TARGETs (Treatment Approaches in Renal cancer Global Evaluation Trial). The Phase III trial demonstrated a statistically significant doubling of PFS and longer overall survival (hazard ratio=0.72 for sorafenib over placebo) in patients treated with sorafenib relative to placebo treatment (Nexavar prescribing information, 2006) (Escudier *et al*, 2005).

Here, we present an analysis of the efficacy and safety of sorafenib monotherapy in a cohort of patients with progressive advanced melanoma.

## MATERIALS AND METHODS

### Patients' characteristics and study design

This Phase II, placebo-controlled RDT was conducted at five centres in two countries (four centres in the US, one centre in the UK). Enrolment began on 25 September 2002. All patients participating in the RDT provided written, informed consent. The trial protocol received institutional ethics committee approval at each participating centre, and was conducted in accordance with Good Clinical Practice guidelines and the Declaration of Helsinki.

The design of this RDT, including patients' details and inclusion and exclusion criteria, has been described previously (Ratain *et al*, 2006). Briefly, eligible patients had histologically or cytologically confirmed, progressive advanced unresectable, or metastatic cancer. Sorafenib was initially administered to all patients in a 12-week, open-label, run-in period using continuous oral dosing at 400 mg twice daily (b.i.d.). After the 12-week run-in period, disease status was assessed based on change in bidimensional tumour measurements from baseline. Patients with ⩾25% tumour shrinkage continued to receive sorafenib open label until they experienced disease progression or unacceptable toxicity, whereas patients with progressive disease (PD; ⩾25% tumour growth or other clinical evidence of progression) were discontinued. Those patients who had an unconfirmed change in tumour size of <25% were randomised in a double-blind fashion to receive either sorafenib 400 mg b.i.d., continuously, or matching placebo from week 12 onwards. Patients with progression of disease – defined as a change in bidimensional tumour measurement from randomisation of ⩾25%, or clinically assessed progression – at any time after randomisation, were unblinded. Progressors from the placebo group were given the opportunity to crossover to sorafenib, whereas those who progressed while on sorafenib were discontinued. In this analysis, we evaluated the efficacy and safety of sorafenib monotherapy in a cohort of patients with progressive advanced (ie unresectable or metastatic) melanoma, who participated in this Phase II RDT.

### Efficacy and safety assessments

As described previously (Ratain *et al*, 2006), the primary end point was the percentage of patients remaining progression free at 12 weeks postrandomisation. Secondary endpoints included PFS after randomisation, overall PFS, tumour response rate and safety (Ratain *et al*, 2006). This analysis will focus on the changes from baseline in bidimensional tumour measurements after the run-in phase, tumour responses for the entire treatment period, PFS and safety findings for a cohort of patients with advanced melanoma.

Tumour response was assessed at 12 weeks, and every 6 weeks thereafter, using standard bidimensional measurements in accordance with WHO guidelines for partial response (PR), stable disease (SD) and PD. Objective responses (ie minor response and PR only) were confirmed at least 4 weeks after the original documentation.

Safety was assessed for the entire treatment period (run-in plus randomisation). All patients who received at least one dose of study drug were evaluable for safety. Safety assessments were performed every 3 weeks during the run-in and randomised phases, and every 4 weeks thereafter. Toxicities were graded according to the National Cancer Institute Common Toxicity Criteria version 2.0 (NCI-CTC v2.0), and relationship to study drug was recorded.

### Biomarkers: BRAF and RAS oncogenes

DNA extracted from the tumour biopsies of patients was screened for the presence of oncogenic *BRAF* and *RAS* mutations. Owing to the majority of oncogenic *BRAF* mutations in melanoma patients are likely to occur within the kinase domain (eg the prevalent V600E *BRAF* mutation), exon 15 was analysed first. Exon 11 of *BRAF* was also screened for the presence of common mutations in the glycine-rich loop. In addition, exons 2 and 3 of *NRAS* (University of Pennsylvania and Royal Marsden Hospital Melanoma Unit) and *KRAS* (Royal Marsden Hospital Melanoma Unit) were screened for common oncogenic mutations. Fresh tumour samples, obtained from patients at The Royal Marsden Hospital Melanoma Unit and the University of Pennsylvania, were snap-frozen and stored at −80°C until use. Genomic DNA from snap-frozen samples, or from paraffin-embedded blocks, was then extracted by lysing tumour samples with proteinase K and tissue lysis buffer, and treatment with RNAse to eliminate residual RNA. The extracted genomic DNA was then purified on a silica-gel membrane column. *BRAF* and *RAS* exons of interest were then amplified by the polymerase chain reaction (PCR) using the following primers, under optimised conditions:



Polymerase chain reaction products were then purified by agarose gel electrophoresis, and automated dideoxy DNA sequencing was performed using the primers that were used for the amplification step and Big-Dye Terminator RR mix. DNA sequences were analysed using the Sequencher 4.2.1 programme.

### Statistical analysis

The PFS attributable to sorafenib was estimated by combining information from the various treatment groups and treatment periods. All patients contributed to the estimate of PFS for the first 12 weeks of therapy. The estimate of PFS for the first 12 weeks was combined with an estimate of PFS after 12 weeks, the latter assuming a patient was alive and progression free at 12 weeks. Progression-free survival was estimated after 12 weeks as a weighted average of group-specific PFS for the two groups (ie open-label and randomised groups). This methodology has been fully described previously (Ratain *et al*, 2006).

## RESULTS

In total, 502 patients with a variety of tumour types, including RCC and melanoma, were enrolled in this RDT; 501 of these patients received sorafenib. This report focusses on the 37 treated patients who had progressive advanced melanoma at the time of study enrolment. The baseline characteristics of these patients are shown in Table 1. Forty-one percent of these patients had an Eastern Cooperative Oncology Group performance status (ECOG PS) of 0, and 57% of patients had an ECOG PS of 1. At baseline, 70% of these patients had failed at least one prior systemic therapy, and 27% had also received prior radiotherapy.

### Twelve-week response assessment

The 12-week sorafenib run-in phase was completed by 33 (89%) of patients with advanced unresectable or metastatic melanoma. The four patients who discontinued before the 12-week assessment all withdrew because of adverse events: three had dermatological events of grade 3 in severity (skin toxicity, *n*=1; plantar–palmar erythema, *n*=2), one had a grade 4 cerebral embolic event.

At week 12, investigator-assessed bidimensional tumour measurements were available for 34 (92%) of the 37 patients. One patient (3%) achieved tumour shrinkage ⩾25% compared with baseline and consequently continued with open-label sorafenib treatment. This patient had an overall duration of treatment of 16 weeks, and PFS of 15 weeks. This patient had three measurable lung metastases. The first lung metastasis showed 75% shrinkage after 106 days of treatment and 84% shrinkage after 136 days. The second metastatic lung lesion had shrunk by 80% after 106 days of treatment and had disappeared by 136 days. The third lung metastasis had completely disappeared after 106 days' treatment. Six patients (16%) had tumour measurements that remained within 25% of baseline levels. The first patient had SD after 89 days of treatment; there was no change in tumour size until PD was proven after 194 days of treatment. The second patient had SD after 79 days' treatment; there was a 5.9% change in tumour size. After 115 days of treatment this patient was proven to have PD. However, at a confirmatory scan after 176 days this patient had SD again. After 428 days this patient was considered to have PD by clinical judgment, but a scan at 512 days revealed that the patient still had SD. The third patient developed SD after 85 days of treatment (24% change in tumour size), was considered to have PD after 113 days (53% tumour growth), but had re-stabilised after 184 days' treatment (9.9% tumour shrinkage). This patient finally progressed after 249 days' treatment. The fourth patient had SD after 96 days' treatment (0.6% change in tumour size) and was still stable after 138 days (6.4% tumour shrinkage). At 404 days this patient still had SD (4.6% tumour shrinkage), but then progressed after 453 days. The fifth patient with SD had progressed after 81 days on treatment, while the sixth patient had SD after 84 days treatment (15% tumour shrinkage), and at day 124 (8.8% tumour growth) but had PD confirmed after two scans after 237 (2.9% growth) and 321 days' (20.6% growth) treatment.

The patients with SD at week-12 were then randomised: three received placebo and three received sorafenib. Twenty-seven patients (73%) had PD (ie tumour growth ⩾25% or other clinical evidence of progression), and were discontinued.

### Antitumour activity: randomised phase

Although all three patients with melanoma randomised to sorafenib progressed at 12 weeks postrandomisation (24 weeks from initiation); only two were discontinued. The third patient was considered by the investigator to be deriving clinical benefit and was, therefore, continued on sorafenib monotherapy. At 12 weeks postrandomisation, all three melanoma patients who received placebo had progressed and, therefore, were crossed over to sorafenib monotherapy in accordance with the study protocol. After crossing over, the three patients had further disease progression at 11, 15 and 22 weeks, respectively. The patient who progressed after 15 weeks remained on sorafenib for a total duration of 73 weeks because, in the opinion of the investigator, the patient was continuing to derive clinical benefit.

### Antitumour activity: entire treatment period

At the end of the entire treatment period, the confirmed investigator-assessed best responses (WHO criteria) to sorafenib were 19% SD, 62% PD and 19% unevaluable. The median PFS for the entire treatment period, based on investigator-assessed data, was 11 weeks (*n*=32; range 9–12 weeks). The six randomised melanoma patients with SD had overall PFS durations of 16 (*n*=3), 18 (*n*=1), 28 (*n*=1) and 34 (*n*=1) weeks. The median time to disease progression for all melanoma patients over the entire treatment period was 11 weeks (*n*=30; range 9–13 weeks).

### Safety

Safety was assessed across the entire treatment period for all 37 patients with advanced melanoma. The most common treatment-emergent adverse events, regardless of attribution, were fatigue (81%); pain (73%); gastrointestinal adverse events, including diarrhoea (51%) and constipation (46%); and dermatological reactions (dermatology/skin – other, 49%; alopecia, 38%; HFS, 35%). The majority of these events were NCI-CTC v2.0 grades 1–2. For example, seven patients (18.9%) had grade 1 alopecia and a further seven (18.9%) had grade 2 alopecia; none had grade 3, 4 or 5 alopecia. Grade 3/4 hypertension was observed in 14% of patients. Serious adverse events were reported in 51% of the melanoma patients, and were attributed mostly to disease progression rather than study drug. Hypertension was not reported as a serious adverse event, and none of the melanoma patients discontinued therapy because of hypertension. Adverse events of any grade attributed by the investigator to study drug (ie drug-related adverse events) were reported in 89% of patients (Table 2). The most common drug-related adverse events among melanoma patients were dermatological (rash/desquamation, 51%; HFS, 35%; and alopecia, 35%); gastrointestinal (diarrhoea, 32%; and stomatitis/pharyngitis, 22%); or constitutional (fatigue, 43%) (Table 2). The majority of drug-related adverse events were grades 1–2 in severity. In total, 8% of patients experienced serious drug-related adverse events (all ⩾grade 3). No grade 4 drug-related adverse events were reported. Six patients required dose reductions because of drug-related dermatological adverse events. Dose interruptions were reported in 12 patients, of which 10 were due to drug-related adverse events.

### Biomarkers

**Figure 1:**
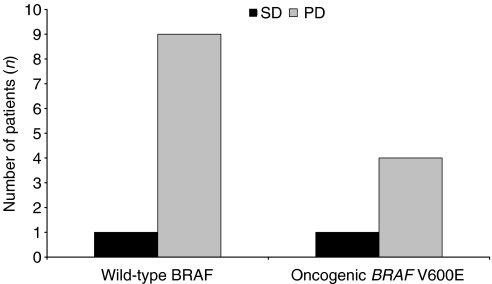
*BRAF* mutational status of advanced melanoma patients is not associated with disease status (status of 15 patients evaluable for response). These patients were also evaluated for oncogenic *BRAF* mutations within exon 11 and oncogenic *NRAS* and *KRAS* mutations. One oncogenic *NRAS* (61K) mutation was found. No other oncogenic *NRAS* or *KRAS* mutations were detected. Two patients were unevaluable for response (*n*=1 wild-type BRAF; *n*=1 *BRAF* V600E) and are not included in the above histogram. DNA could not be extracted from a further five biopsies. The mutational status of the biopsies from five patients was, therefore, not determined (*n*=3 PD; *n*=2 SD).

DNA was successfully extracted and screened from 17 of 22 tumour biopsies. Six biopsies had oncogenic V600E *BRAF* mutations within exon 15, while the remaining 11 had wild-type *BRAF*. Of the six V600E *BRAF*-positive tumour biopsies, four were obtained from patients with PD, one from a patient with SD and one from a patient who was unevaluable for response. Only one wild-type *BRAF*-positive tumour biopsy was derived from a patient with SD, nine were from patients with PD, and one from an unevaluable patient. *BRAF* mutational status data for 15 patients who were evaluable for response are shown in Figure 1. No oncogenic *BRAF* mutations were identified in exon 11 in any of the tumour biopsies evaluated, and only one oncogenic *NRAS* (61K) mutation was detected. No other oncogenic *NRAS or KRAS* mutations were identified.

## DISCUSSION

The efficacy and safety of sorafenib monotherapy were evaluated in patients with advanced melanoma, who participated in a large Phase II RDT involving patients with several advanced solid tumour types (Ratain *et al*, 2006). An analysis focussing on melanoma patients was performed in the light of evidence supporting a role for increased signalling through RAF/MEK/ERK in the onset and progression of melanoma (Davies *et al*, 2002; Chang *et al*, 2004; Garnett and Marais, 2004), and the prevalence of oncogenic V600E *BRAF* mutations in melanoma biopsies (Brose *et al*, 2002). Recent preclinical evidence, showing that blocking V600E *BRAF* expression promotes apoptosis in human melanoma cells, provided a rationale for targeting signalling through RAF/MEK/ERK in the treatment of melanoma (Hingorani *et al*, 2003; Karasarides *et al*, 2004). Despite this rationale, the data in this study at the 400 mg b.i.d. dose studied were disappointing, and are most consistent with a conclusion that the drug has little or no activity as a single agent in this disease.

In human xenograft models, sorafenib has been shown to act on tumour cells to exert an antiproliferative effect, and on endothelial cells of the tumour vasculature to inhibit angiogenesis (Wilhelm *et al*, 2004). Sorafenib also induces apoptosis in several human cancer cell lines (Rahmani *et al*, 2005; Yu *et al*, 2005), including melanoma cells (Panka *et al*, 2006b). Sorafenib downregulates Mcl-1 protein levels in a time- and dose-dependent manner to induce apoptosis in renal, colon and breast tumour lines (Rahmani *et al*, 2005; Yu *et al*, 2005). This effect involves enhanced proteasomal degradation of Mcl-1, which could be the consequence of RAF-1 inhibition. Finally, sorafenib induces caspase-independent apoptosis in A2058 and SKMEL5 melanoma cell lines (Panka *et al*, 2006a). However, the multiple molecular targets of sorafenib, and its dual effects on the tumour cell and the vascular endothelium, make it difficult to determine the mechanism of effect of this multikinase inhibitor in different tumour types, particularly in the absence of validated biomarkers.

In the present analysis, sorafenib monotherapy was well tolerated. Seven patients had PFS lasting between 16 and 34 weeks, although it is not clear whether or not this was an effect of sorafenib. Restabilisation of disease was also observed in one patient who was crossed over to sorafenib after progressing on placebo during the randomised 12-week treatment phase. This patient received sorafenib for a total of 73 weeks. There was no apparent relationship between the presence of an oncogenic V600E *BRAF* mutation within exon 15 and the modest antitumour activity of sorafenib monotherapy. This latter observation is consistent with the lack of a clear relationship reported in another sorafenib trial (Eisen *et al*, 2005). Larger randomised clinical trials are ongoing and will more definitively explore the relationship between response and *BRAF* mutational status.

Despite showing little activity as a monotherapy in this RDT cohort, recently published observations suggest that sorafenib may enhance the antitumour activity of carboplatin and paclitaxel against melanoma (Flaherty *et al*, 2004). In an expanded Phase I study predominantly in melanoma patients, this combination induced one complete response (<1%), 27 PRs (26%) and 61 SDs (58%) in patients with advanced melanoma (Flaherty *et al*, 2004). It is possible that these findings may be due to sorafenib's inhibition of Raf kinase, a known mediator of taxane resistance (Britten and Klein, 2000). In another Phase I trial, the combination of sorafenib with dacarbazine induced a PR in three, and SD in five of 10 evaluable patients with advanced melanoma (Eisen *et al*, 2005). Further investigations are warranted to determine whether sorafenib may significantly increase response to chemotherapy and prolong PFS in patients with advanced melanoma.

Given the preclinical and clinical evidence supporting a role for oncogenic *BRAF* in driving melanoma progression, it is unclear why sorafenib 400 mg b.i.d. did not demonstrate significant activity as a monotherapy in advanced melanoma patients. It is conceivable that this lack of effect is due to insufficient concentrations of sorafenib being achieved within the plasma, and more importantly within the tumours of these patients. The IC_50_ of sorafenib in humans is approximately 5 *μ*M (Clark *et al*, 2005), and may not have been achieved in these melanoma patients. However, pharmacokinetic trials of sorafenib monotherapy have demonstrated steady-state concentrations consistently over the IC_50_ of 5 *μ*M for the recommended dosage of sorafenib (Clark *et al*, 2005). Therefore, further studies are required to investigate this possibility. It is plausible that proliferation of melanoma cells could be driven by an alternative signalling pathway, after signalling through RAF/MEK/ERK has been blocked. A further possibility is that a feedback mechanism could negate the effect of sorafenib in these melanoma patients. This contention is supported by the recent observation that sorafenib administration is associated with increased RAF-1 phosphorylation at Ser338 in human melanoma and other tumour cell types (Adnane *et al*, 2005). However, it remains to be determined whether such a feedback mechanism impairs clinical response to sorafenib monotherapy in melanomas with V600E *BRAF* mutations.

In conclusion, sorafenib is well tolerated but has little or no antitumour activity in advanced melanoma patients as a single agent at the dose evaluated (400 mg b.i.d.). Ongoing trials in advanced melanoma are evaluating sorafenib combination therapies.

## Figures and Tables

**Table 1:** Baseline characteristics for all treated patients with advanced melanoma

**Characteristics**	**Patients (*n*=37)**
Gender (*n* (%))	
Male	23 (62)
Female	14 (38)
Median age (years (range))	53 (18–85)
	
*ECOG PS (n (%))*	
0	15 (41)
1	21 (57)
2	0 (0)
3	1 (3)
	
*AJCC stage at study entry (n (%))*	
III	4 (11)
IV	21 (57)
	
*Most common sites of disease (all lesions)*^a^ *(n (%))*	
Lung	24 (65)
Lymph node	20 (54)
Liver	12 (32)
Adrenal	8 (22)
Median duration of disease^b^ (years (range))	2.7 (0.2–12.9)
	
*Prior therapy (n (%))*	
Systemic anticancer therapy	26 (70)
Surgery	36 (97)
Radiotherapy	10 (27)
	
*Number of prior systemic regimens*	
None	11 (30)
1	13 (35)
2	8 (22)
⩾3	5 (13)

AJCC=American Joint Committee on Cancer; ECOG PS=Eastern Cooperative Oncology Group performance status.

a All target and nontarget lesions occurring with a frequency ⩾20%.

b Years from initial diagnosis to first study treatment.

**Table 2:** Incidence of drug-related adverse events reported in ⩾10% of all treated patients (*n*=37)

	**All grades**	**Grade 3^a^**
**Adverse event**	***n* (%)**	***n* (%)**
Any event	33 (89.2)	12 (32.4)
		
*Cardiovascular*		
Hypertension	6 (16.2)	5 (13.5)
		
*Dermatology*		
Rash/desquamation	19 (51.4)	2 (5.4)
Hand-foot skin reaction	13 (35.1)	4 (10.8)
Alopecia	13 (35.1)	0 (0.0)
Flushing	4 (10.8)	0 (0.0)
Other	12 (32.4)	0 (0.0)
		
*Constitutional symptoms*		
Fatigue	16 (43.2)	0 (0.0)
Weight loss	6 (16.2)	0 (0.0)
		
*Gastrointestinal*		
Diarrhoea	12 (32.4)	0 (0.0)
Anorexia	5 (13.5)	0 (0.0)
Stomatitis/pharyngitis (oral/pharyngeal)	8 (21.6)	0 (0.0)
Nausea	5 (13.5)	0 (0.0)
Other^b^	5 (13.5)	0 (0.0)
Constipation	4 (10.8)	0 (0.0)
		
*Neurology*		
Neuropathy – sensory	5 (13.5)	0 (0.0)

a No grade 4 drug-related adverse events were reported.

b This included: sore gums (grade 1; *n*=1), ulcers on gums (grade 2; *n*=1), mouth soreness (grade 1; *n*=1), and one patient with intermittent stomach upset (grade 1), indigestion (grade 1), and diarrhoea (grade 2).
